# Children’s influence on wellbeing and acculturative stress in refugee families

**DOI:** 10.1080/17482631.2018.1564517

**Published:** 2019-01-29

**Authors:** Disa Bergnehr

**Affiliations:** a Department of Teacher Education, University of Borås, Borås, Sweden; b Department of Social Work, Jönköping University, Jönköping, Sweden

**Keywords:** Refugee families, immigrant parenthood, children’s agency, wellbeing, acculturative stress

## Abstract

**Purpose:** This paper examines intergenerational, interdependent and contextual aspects of wellbeing and acculturative stress in refugee families during resettlement. Particular focus is placed on how children influence their parents. **Method:** The study is based on interviews with and diary notes from Middle Eastern parents and children residing in Sweden. **Results:** Analyzes of the narratives show how the direct and indirect influence of the child affects the parents in both negative and positive ways. Acculturative stress follows from unexpected and undesired migration outcomes, such as parent–child conflicts and low school achievement. Such strains add to other hardships refugee families face, for instance, unemployment, welfare dependence, poor housing, and insufficient mastery of the majority language. However, acculturative stress can be alleviated by the children’s educational success, and reciprocal practices of love and caring including helping out with chores and supporting each other in different ways. **Conclusions:** Children's agency has significant effects on parents’ wellbeing, as wellbeing is accomplished in and through relationships with others.

## Introduction

During the past two decades, Swedish society has received a substantial number of immigrants, many of them refugees. At present, 23% of all children under 18 are of foreign origin, that is, they were born abroad or both their parents were born abroad. Common birth nations are Syria, Iraq, and Somalia (Statistics Sweden, 2017a). Refugee families in Sweden face linked difficulties such as unemployment, scarce financial resources, welfare dependence, insufficient mastery of the majority language, and educational failure (Bergnehr, 2015; FORTE, 2017; Statistics Sweden, 2017b). Compared to natives, immigrants are more likely to experience poor housing, violence, health issues, and a lack of social support (Bask, 2005).

Immigrant families have to adjust to the new societal context and the challenges that migration often entails. Moreover, they may have to adapt their family life to new cultural ideas (Bergnehr, 2016; Kim, Conway-Turner, Serif-Trask, & Woolfolk, 2006; Liamputtong, 2006; Wu, 2011). Intergenerational conflicts and discrepancies are evident among natives as well as immigrants, but migration can cause particular parent–child tensions. Parents’ desire to maintain traditions of their origin may conflict with the child’s desire to acquire the values of the new context (Berry, 2007; Kuczynski, Navara, & Boiger, 2011), and there is a risk that severe strains in the family will evolve into psychological problems for the parent as well as the child (Awad et al., 2013). The challenges associated with resettlement in a new country can lead to “acculturation stress” (Berry, 2007, p. 74), that is, stressors caused by experiences due to migration, and manifested by health issues such as anxiety, uncertainty and depression.

However, conflicting intergenerational gaps do not always evolve. Rather, children and parents often adapt to new values and maintain old ones in tune with each other (Kuczynski et al., 2011). Feeling at home in both the new and old culture creates a sense of belonging and connectedness to the family and across generations as well as to the societal context in which one resides (Bergnehr, 2017). It has been suggested that immigrant youth and parents who jointly seek to adapt to the majority society, while maintaining parts of their cultural heritage, experience greater wellbeing than do those who tend to reject either the habits of their origin or the new culture (Berry, 2007; Berry, Phinney, Sam, & Vedder, 2006). Thus, it is important to study not only intergenerational conflicts in migrant families, but also how family relations and reciprocal support contribute to wellbeing and positive migration outcomes (Dimitrova, Bender, & van de Vijver, 2014). Overall, migrant family life and parent–child relationships have been underexplored (Dimitrova et al., 2014), and research is needed on how parents and children and their relationships undergo changes during resettlement (Kuczynski et al., 2011; Sam, 2014). Scholars have proposed that research should continue to explore immigrant family dynamics and intergenerational relationships (Berry, 2007; Foner & Dreby, 2011). In particular, our knowledge about children’s contribution to family acculturation is scant (Sam, 2014), although there is work investigating bidirectional influences in immigrant families, for instance associations between children’s linguistic and cultural competence and parents’ socio-cultural adaptation (Titzmann & Gniewosz, 2018). However, little has been done to explore how immigrant parents react to stress caused by their children (Sam, 2014). Also, to my knowledge, research is scant on how children’s practical and emotional support contributes to parents’ wellbeing in migrant families.

The purpose of this study is to explore the intergenerational interdependence of wellbeing and acculturative stress in refugee families during resettlement, with particular focus on how children influence their parents. The central research question is how children influence their parents, directly by their actions and indirectly by being a body to protect, nurture and foster, in both negative and positive ways. The study is based on interviews with and diary notes from Iraqi and Syrian families residing in Sweden. To broaden our understandings of wellbeing, we must look at the ways in which societal structures play a role in health inequalities (Stoppard, 2000) and in the accumulation of acculturative stress. By regarding contextual aspects, and utilizing data such as interviews and diary notes, this study and it’s analyzes of narratives can widen our understandings of micro- and macro-processes and how they intertwine (e.g., Daiute & Lightfoot, 2004; Freeman, 1993).

## Theoretical framework

This study is based on the assumption that children directly and indirectly influence their parents’ acculturation and wellbeing. Children influence their parents through their actions and intents, but also by being children whom parents aim to protect, nurture and train (cf., Ruddick, 1989/1995). The child is connected to certain cultural ideals about what entails a prosperous childhood and good parenting, and these ideals influence parents’ practices and notions (Bergnehr, 2008). Parenthood means both strains and rewards for parents; it involves physical and mental hardships as well as love, intimacy and connectedness (Bergnehr, 2008; Palkovitz & Sussman, 1988). However, children’s influence on family life and the implications of this influence have generally not been theorized or explicitly explored to the same extent as parents’ influence on their children (Kuczynski & De Mol, 2015).

This study finds inspiration in theory that emphasizes children’s influence in their social milieu (Kuczynski & De Mol, 2015). Because wellbeing is experienced and contingent on relationships with others (Mayall, 1996; Watson, Emery, Bayliss, Boushel, & McInnes, 2012), children as well as adults both contribute to each other’s wellbeing. Social relational theory offers ways to conceptualize and scrutinize the agency of the child in child-parent relationships (Kuczynski & De Mol, 2015). Agency—being an agent—is perceived as an enduring characteristic that all humans are born with: a person cannot be a non-agent. A person can restrain from taking action, but passivity itself is also an action. Thus, the individual is continuously co-constructing the social world and influencing others with her agency (Kuczynski & De Mol, 2015).

Besides acculturative stress and wellbeing, concepts central to this study are: bidirectional influence and interdependence (Kuczynski & De Mol, 2015; Kuczynski & Knafo, 2014). Bidirectional influence emphasizes the co-creation of intergenerational relationships: parents and children react to each other’s direct and indirect behaviour and being/body; they interpret each other, and confirm, oppose and negotiate each other’s conduct and demeanour. Children and parents continuously adjust to one another and affirm values and ways of acting, but conflicts and disparate views are natural elements of family life and can be the fuel for development and qualitative change. Change characterizes the child–parent relationship. Both parties develop as they grow older, gaining new experiences and facing new contextual restraints and possibilities, which require continuous adaptation (Kuczynski & De Mol, 2015; Kuczynski & Knafo, 2014).

For immigrant families undergoing resettlement, change and adaptation are particularly pertinent. These families often face other challenges than the native population, such as low socioeconomic status (e.g., Bergnehr, 2015; FORTE, 2017). Their wellbeing and psychological health have to be understood in the light of these particular circumstances. Wellbeing is experienced and affected by social relationships, but also by the societal context in which these relationships are embedded (Stoppard, 2000). Adverse conditions, such as poverty, discrimination, loss, domestic violence, trauma, and more, can result in excessive strains that cause harmful stress and, ultimately, illness (Kessler, 1997).

## Methods

The analyzes are based on individual interviews with parents and children, as well as diary notes from the children. Refugee families from Iraq and Syria with a residence permit in Sweden were contacted and informed about the research projects through the schools. Schools also provided rooms in which the interviews were conducted. Participation was voluntary, including the right to withdraw consent to participate at any time. The interviews were recorded, and an interpreter provided assistance during the interview sessions. The diary notes were translated from Arabic to Swedish, and the interviews were transcribed verbatim. When presented here, the names of the participants are pseudonyms and personal details have been deleted or changed to prevent identification.

Prior to data collection, the Regional Ethics Board approved the projects (dnr 2013/458–31, dnr 2016/4–31). Research on minority groups whose lives are comparatively disadvantaged must be conducted with sensitivity so as not to produce categorical descriptions. We aim to broaden the knowledge based on immigrant family life, and the data have the potential to contribute new understandings to the public and political discourse. The data also help to give voice to otherwise disregarded and silent/silenced perspectives. The participants expressed gratitude for being given an opportunity to share their reflections on everyday life—their joy as well as despair. Positive aspects of participation in a research study may thus be self-refection and the experience of being listened to. However, it is important to acknowledge the precariousness of posing potentially sensitive interview questions to persons who have experienced and are experiencing adverse and insecure circumstances. The research team offered professional help to participants should any psychological problems emerge during or after data collection.

### Study participants

The data were collected in two research projects on migrant family life. The families resided in disadvantaged areas. They had arrived in Sweden 0.5–9 years ago and were legal residents of the country. Most of the parents were unemployed, and most families were welfare dependent. Those who were unemployed were studying the Swedish language or had trainee positions. A couple of the mothers were on parental leave. A few of the men were employed in occupations that resembled their work prior to migration. The men’s educational experience varied from a few years of primary school to higher education. Some women had attended higher education and had been in paid employment in their country of origin, but the majority had a secondary or upper secondary education, and little work experience due to having married young and cared for home and children.

One part of the sample constitutes 11 individual interviews with mothers originating from Iraq. In the interviews, they referred mainly to their child in primary school. The Parent Development Interview Guide (Aber, Slade, Berger, Bresgi, & Kaplan, 1985) was used, focusing on the parent–child relationship and parenting. The interviews lasted for 1.5–2 hours.

The other part of the sample constitutes 15 families originating mainly from Syria. The participants were mothers, fathers and adolescent children. The participants in the sample took part to varying extents—some wrote diary notes or participated in the interview only, others took notes and participated in the interview. The analyzed sample consists of: 10 interviews with fathers, 10 interviews with mothers, 9 interviews with children (6 girls and 3 boys), and diary notes from children (10 girls and 6 boys). The children were 12–15 years old.

The diary notes were made during a period of two weeks—one ordinary school week and one week of school holidays. In the diary, the participants were encouraged to state their activities and social company during the day, including any joyful, unpleasant or upsetting events. The interviews with children and parents were centred on questions about everyday life, such as their occupation during the day, family activities during weekends and holidays, their experience of school or language studies/training, plans and wishes for the future, and present challenges and concerns. The parents were asked additional questions about their parenting and the parent–child relationship.

The child interviews lasted for 15–40 minutes, the parent interviews for 45–80 minutes. A parent was present during the children interviews, with a few exceptions, but did not interfere verbally. Two siblings were interviewed together. One woman was present when her spouse was interviewed, and one adolescent girl during the interview with her mother. Interview data are co-constructions of the participating parties (Mishler, 1986), in this case the study participant, the interviewer, the interpreter, and, in some interviews, a parent/sibling/daughter/spouse. Unavoidably, the context of the interview (both social and societal) has an effect on what is said and unsaid.

### Analytical procedure

The interviews and diary notes were analyzed as talk and text that exemplify how migrant parents and children explicate themselves and their relationships. A person’s description and sense of a relationship are based on previous experiences of the relationship, on present circumstances, and on expectations concerning the future (Kuczynski & De Mol, 2015). In the data for this study, the parents and children connected past, present and future in their aim to describe, explain and understand themselves and others (cf. Freeman, 1993). Their relational history was evident in their narration, as was the influence of the societal context on their relationships (Bergnehr, 2017).

Typical of any interview sample, and also of diary entries, is that some participants are more elaborate in their answers, while others keep their discourse short (e.g., Bergnehr, 2008). During the interviews, the children’s answers were generally brief compared to the parents’. Also, the data contained three times as many parents. Consequently, quotes from the parents’ narratives dominate the results section.

The interview transcripts were scrutinized and coded for sequences in which interdependence and bidirectionality in the child–parent relationship emerged. The sequences chosen for further analyzes involved direct actions that presumably affect others’ wellbeing as well as talk about how the child indirectly—by being a body to be protected, nurtured and trained (Ruddick, 1989/1995)—influences the parents. Accounts of wellbeing were defined as involving: positive emotions, a sense of engagement and inclusion in the social surroundings, relationships that provide support, meaning-making involvement in activities, and achievement (Seligman, 2011). In the analytical procedure, wellbeing was further defined as indications of: a maintained state of being generally comfortable despite brief moments of distress. The opposite of wellbeing, in this study conceptualized as acculturative stress, entailed indications of a prolonged state of discomfort, unhappiness and/or recurrent worries ensuing from migration, which came across as overshadowing everyday life.

The sequences were categorized into two themes: unfavourable influence and favourable influence. Unfavourable influence contained discursive articulations indicating concerns and stress on the part of the narrator. Favourable influence indicated the opposite, that is, contentedness and wellbeing.

As humans, we influence each other in both positive and negative ways. Trying to define when the influence is beneficial or not can be a delicate matter. For instance, conflict and contradiction in the parent–child relationship are not necessarily detrimental. It has been argued that they precipitate change, development and learning (Kuczynski & Knafo, 2014). But while emotional and practical support and the sense of connectedness are generally advantageous for wellbeing, unremitting stress and concern about the other, or reoccurring conflicts, may have negative implications. This study illuminates both negative and positive influences in the child–parent relationship, as they are reflected in the parents’ and children’s narratives.

## Results

The outline of the results section is based on the two analytical themes: unfavourable influence and favourable influence—conceptualized as *interdependent acculturative stress* and *reciprocal wellbeing*, respectively.

### Interdependent acculturative stress

Migration, in particular for refugees, occurs in reaction to harsh and often life-threatening circumstances. Conflict and war mean unique difficulties for the parents to protect children from harm, and to provide a favourable environment in which they can grow and prosper. The desire to provide more prosperous futures for their children can thus trigger the decision to migrate (Sam, 2014). A recurrent narrative among the parents in this study was that their children’s welfare was the reason they decided to migrate. Their worries and concerns for the wellbeing of their offspring overshadowed life, and a decision had to be made. This is an example of indirect child influence; the parents totally changed their lives on behalf of their children’s welfare. Lea, for instance, said:

We lived 5 years during war in Syria, but then our son turned 18 and had to join the army. The ones who join the army don’t come back, there is no return, and I didn’t want to lose my son, so that’s why we decided to come here. It was a really, really tough decision to make.

In order to protect their son from serving in the military and from the risk of premature death, Lea and her husband chose to migrate with the family intact. However, after arriving in Sweden, the oldest son, due to his age, was granted a temporary residence permit and was not eligible for school during the asylum-seeking process, as were his younger siblings. The idle period of having nothing of significance to do for a year resulted in depression and despair. This also negatively affected his parents. Lea repeatedly, in the interview, brought up her concerns about him, caused by the migration: “I’m so sad and disappointed. Everything I’ve done, I’ve changed my life and migrated, was for the sake of our son. But when we arrive [in the new country], he gets nothing.” This example illuminates the interdependence of wellbeing and acculturative stress, and also how stress is caused by unexpected consequences of immigration. The parents migrated on behalf of their child, but inadvertent consequences caused the child misery and thus also acculturative stress for the parents. It exemplifies the importance to regard contextual aspects when conceptualizing migrant children’s and parents’ wellbeing; stress is caused by rules and regulations in the receiving country that are out of the individual’s control.

Like Lea, Jamal referred to the age of his oldest son as the decisive reason why the family migrated. He, himself, would rather have stayed in Syria, but the family migrated to provide future prospects for the children, and for his son to avoid military service. But life in Sweden brought new concerns and worries, although different ones from those raised by Lea. Jamal talked about Swedish parenting ideals being very different from those in Syria and his feelings of uncertainty about how to act as a parent: “Too much freedom” is allowed children in Sweden. This reasoning accords with previous studies on migrant mothers (Bergnehr, 2016). Jamal’s oldest son, who was in middle adolescence, had appropriated Swedish cultural values that deviated from the family values, Jamal argued. According to him, this deviation may have a detrimental effect on his son’s education and future prospects:

In my country, I knew exactly where he was, at this neighbour or with that person. Here, we don’t know anybody, you don’t know where he is, you don’t know nothing, where he’s at or with whom. We tell him everyday: “We are worried about you, you are not allowed to behave like this, it’s wrong”. He says “Yes”, but then he’ll do just like he pleases. For instance, when he goes out, if I don’t call for him he doesn’t come home. I believe that he must better consider his future, his studies. We came here for his sake, for the children’s sake. [If he fails at school] he has ruined his life, and I have ruined mine.

In Jamal’s narrative, he expressed great concern about his son who disregarded his schoolwork and deviated from family norms. Discrepancies between child and parent thus appear to be one source of acculturative stress; the stress accumulates due to parents’ lack of familiarity with the new cultural context. The following quote from Helena is yet another example of this sort of reasoning, indicating the acculturative stress among the parents that ensues from the direct or indirect influence of their children:

I am worried about her future. I don’t know what will happen. She is going to a new school and I feel really worried about her, because I’m not that good at the Swedish, and I am really worried about the friends she’s going to meet. It’s very difficult to live in a new country.

Helena and Jamal, and others, bring up their lack of knowledge of the Swedish language and the Swedish parenting culture, which make it harder for them to control and know various things about their children. Not knowing and/or being able to speak to their children’s friends, and the friend’s parents, increase the sense of stress and lack of control. Of particular worry is that their children’s socializing with friends will have a negative effect on schoolwork, and thus on the child’s future. Jamal stated, in the interview, that his life was ruined by migration, with few opportunities for employment and upward mobility in the new country. His family migrated for the future of his children. Should his children fail at school and fail to gain employment and self-provision, his ruined life would have been for nothing. The other parents’ discourse is similar to Jamal’s. All emphasize the importance of educational success, and many bring up concerns about their child’s schooling. Refugee parents’ are realistic in their worries; official statistics show considerable differences in achievement between the in-born and foreign-born students (FORTE, 2017).

Motivation is not the only thing affecting a person’s school results. The child’s personality and cognitive disposition affect her relations and being in the world. A father, Jacob, has two adolescent daughters of whom one was having difficulties at her Swedish school, although both of them had done well in Syria. His concerns are exemplified by the following quote:

In Syria, she was doing well [at school], both of them, her and her sister, and it is just one year in age between them. They got great grades, both of them, good results. Obviously, we put a lot of effort, myself and my wife, into helping them with their studies. Obviously, we cannot help them in the same way here, due to our insufficient language skills. I get sad inside because I know how well she was doing in Syria, but not here, no. Why?

The quote from Jacob illustrates the concerns and stress that ensued from migration. Lack of skills in the majority language is brought up here as preventing the child from succeeding at school as well as the parents from supporting their child’s schoolwork. This may add to the stress; the parents are unable to help the child in the ways they had prior migration and wish to do.

There are several examples in the data where parents and children concur in their emphasis on education. In interviews and diary notes, the children bring up concerns when they feel they have difficulties at schools. Elisabeth, for instance, raised concerns about not doing well at school—she wrote in her diary that she worries about the national test, but also that she is studying for the test, i.e., how she uses a strategy for improving her school achievement. Naomi elaborated, in the interview, on why she likes school: “If you can’t read or write you can’t do anything with your life. Like if you don’t succeed at upper secondary education, you can’t get work, because you need this document that you’ve made it.”

Children influence their parents indirectly, and directly by their actions, and vice versa. When life-changing decisions are made, such as migrating to a new country, positive outcomes are anticipated but may be obstructed, causing the child as well as the parent distress.

### Reciprocal wellbeing

Acculturative stress can be prevented or alleviated by achievement, reciprocal care and support, and a sense of connectedness. All parents refer to children’s success at school as something that gives them happiness and content. Lydia, for instance, said: “I want my child to become something acceptable, that’s what’s most important, that he gets a good education.” Jacob, in accordance with other parents, answered the following to a question asking what gives him most joy about his children: “When they achieve at school, that makes me happy.” The parents referred to feelings of joy, happiness, content and relief in connection with their child’s school success. Another example is Amira, who said: “What brings me the most joy, is if my daughter tells me ‘Mum, look at my high points on the school test.’ Good results give me most joy. That makes me feel I have succeeded.” The child’s achievement is connected to parental success, that is, the realization of what the parents desired and anticipated from the migration.

Achievement, social relationships, and a sense of belonging are criteria for wellbeing (Seligman, 2011), and the child’s educational attainment raises hopes for a better future and upward socioeconomic mobility (Bergnehr, 2016, 2017). The children’s discourse concurs with that of the parents. They refer to being contented when they do well at school. For instance, when indicating the “event of the day” in his diary, Gabriel wrote: “I passed the test”, and later on in the week: “I got good results on the test.” Elisabeth wrote in her diary that she enjoys school and her friends there. This accords with what generally emerged in the data produced by the children. Like Adam, who in response to the question “What do you like to do most an ordinary day?” said: “I like school the most…. All my friends are there.” In the children’s narratives, school was depicted as engendering wellbeing in that they experienced joy and fun there. Patricia wrote in her diary the first day after school holidays: “I got up in the morning and I went to school. I got home at 2.30 pm, and I was really, really happy because I have longed for school.” Also, one girl, Anna, answered the diary question about whether anything exciting or fun had happened during the day: “When the teacher, my parents and I had a discussion on my progress at school. That was exciting and fun because then my parents got to know about my achievements.”

To love and care for others and to be loved and cared for are essential for human development and wellbeing. Care is ubiquitously dialogical and bidirectional, and produces a sense of togetherness and belonging (Noddings, 2013). Examples of reciprocal care that children and parents brought up are physical contact, words of love, presents, appreciated meals, showing an interest in the other person’s feelings, ideas and aspirations, and helping out with everyday chores.

In the parent interviews, younger as well as older children were described as caring, loving and attentive to the feelings and needs of the parent. Darah, for instance, reported compassion as a characteristic of her 6-year-old son that she appreciated the most: “He’s got compassion. For example, one day when I sat in the sofa with a headache, he came up to me and asked ‘Mummy, what’s wrong with you?” The narratives from the other mothers in the sample contained similar descriptions. Lea referred to her 19-year-old son, and what she likes about him the most, in the following manner: “I really like his way of noticing when I’m mentally exhausted. Then he comes up to me and hugs me, he feels with me and talks to me.” Mothers appreciated their children’s expressions of care and compassion when they were distressed; presumably these expressions supported their sense of wellbeing. Lydia’s narrative provides yet an example. In her talk about the parent–child relationship, she described how her son expressed his love for her: “For instance, when I hear that my mother and father are ill, they don’t live here in Sweden, then I cry, and then he comes and wipes my tears. He says: ‘Mummy, don’t cry’, and hugs me. That’s love. Also, we talk a lot.”

The bidirectional, interdependent and reciprocal aspects of family life and wellbeing emerged in the interviews. The children’s diaries and interviews contained many examples of how they help their parents with daily chores, such as cleaning, tiding up, fixing food, and playing with younger siblings. These were usually mentioned as activities accomplished together with the parent, such as Alexander depicted in his diary: “Me and my father cleaned up at home”, and Naomi in hers: “I spent the evening with my mother. She had lots of washing to do, so I helped her out.” This was mirrored in the parent interviews, where many mothers, when describing their child and the parent–child relationship, brought up their child’s initiatives to help out. One example is provided by Sofia who talked about her 8-year-old son in the following way: “He is helpful, he wants to help. He always comes up to me and asks: ‘Do you want some help, Mummy? Is there anything I can help you with?’ That makes me happy.” Another example that illustrates both compassion and helpfulness on the part of the child, and the child’s ability to support the parent, is found in the narrative of Jasmine, who referred to her 7-year-old son: “He knows me, and he says to me: ‘Mummy, why are you angry today, why are you sad? Are you tired, Mummy? I can help you with the dishes”’.

Essential ingredients in positive human relationships are caring for others and being cared for (Kuczynski & De Mol, 2015; Noddings, 2013). These data provide multiple examples of care and connectedness in the parent–child relationship. Parents and children brought up the joy of spending time together, such as playing games, watching a movie, having dinner and talking about life, visiting a restaurant, having relatives over, etc. The repeated references to family socializing and the importance of family suggest that the family and the parent–child bond are vital to the recreation of connectedness and belonging during periods of resettlement (see also Bergnehr, 2017), and have significant implications for the wellbeing of parents and children alike.

## Concluding discussion

Children and parents are both contributors to influencing each other’s wellbeing. This study helps to illuminate these processes and of how they take shape in refugee families. It produces further knowledge on forced migrant resettlement, and provides a contrasting picture through its focus on agency contrary to recurrent public and political depictions of refugees, particularly children, as vulnerable victims who are exposed to adversities and discriminating social structures. Though true to some extent, such depictions often ignore the agency of the migrants, and thus their influence on other individuals, their resources, strengths, capabilities, and power to affect their own lives.

The ideas of interdependence and bidirectionality are established in our understanding of human development, but a unidirectional model continues to influence conceptualizations of the parent–child relationship in much research and public discourse (Burman, 2016; Kuczynski & Knafo, 2014). This study emphasizes bidirectionality and the agency of the child. Agency is conceptualized as an intrinsic quality of every human being—a person cannot be a non-agent (Kuczynski & De Mol, 2015). Through direct and indirect influence, the child, by being an agent, affects her parents and their relationship, and consequently, since wellbeing is embedded in social relationships (Watson et al., 2012), her own and her parents’ wellbeing.

The term *interdependent acculturative stress* has been applied to analyze how children’s direct and indirect influence can cause their parents a prolonged state of concern, anxiety and despair. Berry (2007) introduced acculturative stress as being a state of anxiety, uncertainty or depression due to challenges caused by resettlement in a new country. Here, Berry’s term has been combined with Kuczynski’s and others’ dialectial, bidirectional model of socialization and enculturation (Kuczynski & De Mol, 2015; Kuczynski & Knafo, 2014). Acculturative stress is conceptualized as being contingent on interdependent processes, that is, the agency of both children and parents, and the challenges that they face in their separate daily activities (e.g., school and work) and in family life and the parent–child relationship.

The results of this study suggest that many parents migrate primarily for the sake of their children. Those parents who during resettlement experience unexpected and unwanted migration outcomes, such as their child having difficulties at school, or increased parent—child conflicts due to new parenting norms, may face unremitting stress with negative implications for their health and wellbeing. This, in turn, affects their children, and the child–parent relationship. Acculturative stress is an interdependent, bidirectional process that is also influenced by societal structures, that is, opportunities and restraints that children and parents experience in the receiving country.

The concept *reciprocal wellbeing* has been utilized in this study to analyze how parents and children contribute favourably to one another’s wellbeing. Most human relationships contain negative and positive bidirectional influence. Parents, for instance, experience both strains and rewards from parenthood and their relationship to their child (Bergnehr, 2008; Palkovitz & Sussman, 1988, see also Parker, 1995/2005). However, it appears to be more common to investigate intergenerational conflicts and unfavourable influence rather than how relationships are characterized by reciprocal practical and emotional support that, for instance, may help to alleviate acculturative stress. This work is informed by the proposition that wellbeing is accomplished in and through relationships to others (Mayall, 1996; Watson et al., 2012). Added to this are the central ideas of bidirectionality and reciprocity; caring for others and being cared for are essential parts of being human (Kuczynski & De Mol, 2015; Noddings, 2013). Children are in the Western World most often thought about as being cared for, and parents as being the ones responsible for providing the care. However, children also care for family members, and provide practical and emotional support (Tryggvason, 2018). Children’s contribution to parents’ (and other family members’) wellbeing needs acknowledgment without being pathologies and problematized as undesired “parenting” behaviour.

These results suggest that children’s and parents’ wellbeing is closely connected to children’s school achievement. In several cases, parents and children seemed to have similar goals regarding children’s attainment, although their aspirations may be hindered by, for instance, language difficulties. It can take years for a person to learn a new language, and children as well as parents expressed frustration and hinders due to insufficient skills in the majority language. Language problems slowed down the child’s educational progress, and restrained the parents from supporting the child’s learning, for instance by helping out with home-work. Consequently, the families experienced a double-edged limitation in language skills that affected the child’s school achievement. Discrepancies in values and goals due to intergenerational differences in part caused by new parenting norms in the receiving country also appeared in the parents’ narratives. The parents connected potential and actual parent-child value conflicts with an increased risk for school failure.

In Sweden, educational attainment is pertinent to self-provision, upward social mobility, and good health, but immigrant children run a considerably higher risk of school failure compared to natives (FORTE, 2017). Thus, it is only to be expected that school results would be a great cause for concern, and that educational failure could engender acculturative stress, for children as well as their parents. It has been suggested that immigrant children feel great pressure to succeed at school, causing them health problems (Foner & Dreby, 2011). However, the parents’ emphasis on achievement is logical since educational success means the promise of new opportunities. Swedish society must better support immigrant families in their strivings for a prosperous future through their children’s educational achievement. The schooling system needs to be organized in ways that better enable children of foreign origin to succeed.

Expected and desired outcomes of migration have the potential to alleviate acculturative stress. Parents as well as children associated the child’s educational achievement with the sense of content, happiness and success. The narratives also suggest that children contribute positively to their parents’ health status by providing love, care and a sense of connectedness (see also Bergnehr, 2017). The children bring purpose and meaning, joy and hope to their parents. There is a reciprocal relationship between parent and child that emerges in the data, where practices of love and caring, including helping out with chores and supporting each other in different ways, permeate everyday life. This certainly has significant and positive effects on parents’ as well as children’s wellbeing.

One cannot be human without influencing others and thus it is of interest to scrutinize and encourage reflection on how parents and children influence each other. To widen our understandings of human development, of parent–child relationships and of acculturation and resettlement, unidirectional models and sole focus on parents’ influence on their children are preferably avoided. Parents’ *and* children’s agency must be considered in the design of health-promoting initiatives and family support services, in home-school collaboration and social policy reforms. This study contributes with knowledge on refugee children’s direct and indirect influence on their parents’ wellbeing during resettlement. In addition, it illuminates and applies theorizing on children’s agency, bidirectionality and intergenerational interdependence. It proposes a holistic, relational view on children’s agency and influence, where negative as well as positive aspects are acknowledged. Conflict and tension are not necessarily detrimental to the child–parent relationship; it can engender reflection, development and qualitative change for parents as well as children (Kuczynski & De Mol, 2015). One thus has to be careful to state that conflicts in families by definition are bad.

It is common to regard practical and emotional support in the family as something primarily provided by the parents. However, by doing so, one oversees children’s contribution but also the relational, reciprocal nature of human life. This study has provided examples of how children, as agents, influence their parents in varying ways and evidently contribute to decision-making, everyday family life, and their own and their parents wellbeing. The study hopes to inspire future research to focus on children’s agency in relation to other family members and the wider society, and health care staff, educators, social workers and other practitioners to acknowledge children’s influence in their dealings with families.
